# 

**Published:** 1886-02

**Authors:** 


					﻿1886.
\ /	26 ADDITIONAL PACES.
OLD SERIES
Vol.
nev\> *\wgs

Mtwfa
fflEDIGAL«UR»
JOURNAL?
f’ax et	3
'^7XvV4^Scr>rZ\At- z)'
^b,
W.F.WESTMORELAND.I®
H.V.M.MILLER.M.D.LL .D.Jk
JAMES A.GRAY. M.D."
Published By james rharrison&cqs
tflP	____tflTLANTA.GA.
SUBSCRIPTION: $2.50 A YEAR.
				

